# Clinical evaluation of probe capture-based targeted next-generation sequencing in suspected infected pancreatic necrosis: a prospective pilot diagnostic study

**DOI:** 10.1186/s12879-025-12441-w

**Published:** 2025-12-29

**Authors:** Baiqi Liu, Dingcheng Shen, Xintong Cao, Xiaoyue Hong, Chiayen Lin, Rong Guo, Zefang Sun, Caihong Ning, Lu Chen, Yan Yu, Jiarong Li, Gengwen Huang

**Affiliations:** 1 Division of Pancreatic Surgery, Department of General Surgery, Xiangya Hospital, Central South University, Changsha, Hunan Province 410008 China; 2 Division of Hernia and Abdominal Wall Surgery, Department of General Surgery, Xiangya Hospital, Central South University, Changsha, Hunan Province 410008 China; 3 National Clinical Research Center for Geriatric Disorders, Xiangya Hospital, Central South University, Changsha, Hunan Province 410008 China; 4 FuRong Laboratory, Changsha, Hunan Province 410078 China; 5 Changsha KingMed Center for Clinical Laboratory, Changsha, Hunan Province 410221 China

**Keywords:** Acute pancreatitis, Infected pancreatic necrosis, tNGS, mNGS, Virulence factor

## Abstract

**Objective:**

To evaluate the diagnostic performance of probe capture-based targeted next-generation sequencing (tNGS) in suspected infected pancreatic necrosis (IPN).

**Methods:**

A prospective study was conducted among patients with suspected IPN at Xiangya Hospital from December 2023 to May 2025. Blood samples were collected and sent for tNGS, metagenomic next-generation sequencing (mNGS), and culture. For patients with indications for surgical interventions, peripancreatic specimens were simultaneously collected for microbial culture during the initial surgical procedure to confirm the presence of IPN. The clinical performance of tNGS, mNGS and culture were compared.

**Results:**

In 51 patients, blood tNGS demonstrated a significantly higher positivity rate compared to blood culture (54.9% vs. 17.7%, *P* < 0.05), while no statistically significant difference was observed between blood tNGS and blood mNGS (54.9% vs. 51.0%, *P* = 0.683). tNGS demonstrated superior detection of fungi and Gram-negative bacteria. With peripancreatic culture as the reference standard, tNGS demonstrated superior sensitivity (94.7%), accuracy (81.3%), and concordance rate (78.9%) compared to conventional culture, and exhibited comparable performance to mNGS. tNGS also detected antimicrobial-resistance genes in 7 patients, with findings consistent with available phenotypic susceptibilities. Detection of virulence factors (VF) was associated with higher rates of persistent organ failure (POF) (100.0% vs. 57.1%, *P* = 0.009) and mortality (77.7% vs. 11.9%, *P* = 0.022). Firth’s bias-reduced logistic regression identified OF > 7 days as an independent predictor of tNGS positivity (OR 4.259, *P* = 0.040).

**Conclusions:**

Blood tNGS demonstrated diagnostic performance comparable to mNGS in suspected IPN, with potentially greater sensitivity for fungal and Gram-negative pathogens. Its ability to detect VF may offer prognostic insights, but clinical utility requires further validation.

**Supplementary Information:**

The online version contains supplementary material available at 10.1186/s12879-025-12441-w.

## Introduction

Infected pancreatic necrosis (IPN) is a severe complication of acute pancreatitis (AP) associated with high morbidity and mortality [1]. Timely identification of pathogens and the initiation of targeted treatment are essential for improving patient outcomes. However, the diagnosis of IPN remains a significant clinical challenge due to its complex presentation and the potential for severe complications. Traditional diagnostic methods, such as fine needle aspiration for microbiological culture, are not routinely used and are often reserved for selective cases due to the risk of complications and the invasiveness of the procedure. Moreover, the reliance on clinical parameters, such as fever and elevated inflammatory markers, can lead to diagnostic uncertainty, particularly in the absence of gas on contrast-enhanced computed tomography [2, 3]. A promising approach for pathogen detection is metagenomic next-generation sequencing (mNGS), which enables comprehensive analysis of microbial communities in clinical samples. In a prospective study, mNGS demonstrated a diagnostic sensitivity of 95.2% when analyzing blood samples from patients with febrile acute necrotizing pancreatitis (ANP), significantly higher than the sensitivity of blood cultures, which was only 23.8% [4]. Furthermore, adjusting antibiotic therapy based on mNGS results was found to potentially improve the correct usage rate of antibiotics from 18.6% to 81.4% [5]. However, mNGS has limitations, especially for intracellular pathogens, fungi, and low-abundance bacteria in patients previously exposed to antibiotics [6, 7]. Additionally, the high cost of mNGS remains a barrier to its widespread adoption in clinical settings [6, 7]. Therefore, a more sensitive, rapid, and cost-efficient method for diagnosing IPN is urgently needed.

Targeted next-generation sequencing (tNGS) has emerged as a powerful diagnostic tool in infectious diseases, showing superior performance over mNGS in respiratory and central nervous system infections [8, 9]. The rapid turnaround time of tNGS, often delivering results within 6 to 48 h, is particularly beneficial for guiding antibiotic therapy and initiating appropriate treatment strategies in critically ill patients [10]. This is achieved by targeting specific genomic regions (e.g., 16 S rRNA for bacteria, internal transcribed spacer for fungi), which simplifies analysis and accelerates pathogen identification [11]. tNGS has also shown exceptional efficacy in detecting fungal infections and mixed infections, which are increasingly prevalent in critically ill patients [8]. In clinical practice, the prognosis of IPN varies significantly depending on the causative bacterial pathogen, with Escherichia coli infection demonstrating a notably favorable outcome compared to other microorganisms [12]. Fungal infections and multidrug-resistant (MDR) bacterial infections have emerged as critical risk factors for IPN. Although the underlying mechanisms remain poorly understood, one plausible explanation is the differential expression of virulence genes. Existing studies have shown that there were specific bacteria and virulence factors in emphysematous pancreatitis through NGS [13]. Therefore, detection of specific pathogen and virulence genes through tNGS offers a promising avenue for understanding the underlying mechanisms of pathogenicity in IPN.

In this study, we evaluated a blood probe capture-based tNGS workflow for suspected IPN. We compared its diagnostic efficacy and turnaround time with traditional detection methods and mNGS, and analyzed the relationship between VF and prognosis.

## Methods

### Study design

This prospective observational study included patients with AP admitted to Xiangya Hospital, Central South University from December 2023 to May 2025. Study inclusion criteria were (i) AP diagnosed according to the Revised Atlanta classification [14]; (ii) presence of pancreatic necrosis confirmed by computed tomography (CT) scan; (iii) age older than 14 years; (iv) febrile, with temperature ≥ 38.5 °C during the disease course. Patients who were pregnant or who had prior abdominal or retroperitoneal invasive procedures were excluded. The study was approved by the Ethics Committee of Xiangya Hospital, Central South University, China (reference: 202304286). Written informed consent was obtained from all participants aged 16 years or older. For participants younger than 16 years, informed consent was obtained from their parents or legal authorized representatives. This study has been reported in line with the STARD and STROBE guidelines and in accordance with the Declaration of Helsinki [15].

### Sample collection and data acquisition

Blood samples were collected simultaneously for mNGS, tNGS, culture, and other routine biomarkers such as C-reactive protein (CRP), procalcitonin, interleukin-6 (IL-6), interleukin-10 (IL-10), during fever (T ≥ 38.5 °C) [5]. For patients who met surgical indications, peripancreatic specimens were collected for microbial culture during the initial surgical intervention. The data of clinical manifestations, laboratory tests, imaging results, antibiotic usage, and clinical outcomes were extracted from electronic medical records [5].

### mNGS process

The mNGS process was performed as described previously [4]. Genomic DNA was enzymatically fragmented and subjected to library preparation, including end repair, adaptor ligation, purification, PCR amplification, and circularization to obtain single-stranded DNA circles. The qualified libraries were sequenced on the BGISEQ-2000 platform using 50-bp single-end reads. Subsequent bioinformatic analysis was performed with a dedicated pipeline encompassing 32 antimicrobial resistance genes.

### tNGS process

The tNGS process was performed as described previously [16]. Contamination control protocols incorporated strict spatial partitioning to prevent cross-contamination between steps, pre-validating all consumables as nucleic acid-free, and incorporating a no-template control in each batch as a negative control. Nucleic acid was extracted from 300 µL of plasma using the MasterPure DNA & RNA Extraction Kit (KingCreate) following the manufacturer’s operational manual. And the libraries were constructed through RNA transcription for cDNA, DNA fragmentation, end repair, adapter ligation, and PCR amplification. The biotinylated probes used in this study were designed to focus on capturing 1,292 species of bacteria, 517 species of fungi, 1,380 species of viruses, 210 species of parasites, 2,627 resistance genotypes of 86 antimicrobial resistance (AMR) genes, and 64 virulence genes. Then, eight uniquely barcoded libraries were pooled for hybridization and captured using specific biotinylated probes for 2 h after library generation using the MetaCAP Pathogen Capture Metagenomic Assay Kit (KingCreate), following the manufacturer’s instructions. Using the Qsep100 Bio-Fragment Analyzer (Bioptic Inc., Taipei, China) for the quality control of the libraries and showed peaks near 350 bp. While absolute quantification was performed with the Qubit 4.0 fluorometer (Thermo Fisher Scientific, Waltham, MA, USA), and sequencing was performed on an Illumina MiniSeq platform set to 100-bp single end with average of 1 million reads per sample. Environmental and background contamination control followed a predefined strategy that included strict spatial zoning of laboratory workflow, nuclease-free quality control of all consumables and reagents, continuous negative-control monitoring in each batch, and sterile management of personnel and environment. Interpretation of low-biomass taxa was based on predefined thresholds: post-filtered clean reads > 300,000, post-filtering Q20 > 90%, internal reference gene GAPDH with RPhM > 100 or coverage ≥ 0.5, and pathogen-specific reads ≥ 3. Finally, the raw sequencing data were analyzed using the bioinformatics pipeline [16]. Detailed diagnostic workflows, VF panel and a relative cost framework for blood culture, tNGS, and mNGS are provided in Table S1 and Table S2.

### Microbial culture

The fully automated blood culture apparatus was BACTECFX (Becton, Dickinson and Company). Bacterial identification was performed by using the VITEK MS detection system (BioMérieux, Marcyl’Étoile) [5]. A positive culture was defined as the detection of a specific pathogen such as bacteria or fungi in the specimen, while a negative culture was defined as the absence of any organisms growing during the incubation period. Polymicrobial infection was defined as more than one pathogen detected in a sample. Multidrug-resistant organisms (MDRO) infection was defined as microorganisms not susceptible to at least 1 agent in at least 3 microbial categories [17]. Extra-pancreatic organisms detected by blood or drainage samples were recorded when applicable. Extra-pancreatic organisms were adjudicated jointly by laboratory medicine physicians, infection-control specialists, and pancreatology clinicians. Adjudication incorporated the presence of systemic inflammatory response, organ dysfunction, imaging findings suggestive of infection, and clinical improvement following pathogen-targeted therapy.

### Management protocol of AP

This study was a real-world observational trial. The results of NGS were blinded to the multidiscipline teams consisting of pancreatic surgeons, intensive care physicians, gastroenterologists, and interventional radiologists. Participants in the study received treatment that adhered to the guidelines established by the International Association of Pancreatology and the American Pancreatic Association [18]. This included proper fluid resuscitation, prompt enteral nutrition, titration of vasopressors, mechanical ventilation, and renal replacement therapy. Broad-spectrum antibiotics with the ability to penetrate pancreas were administered to patients with febrile ANP when the body temperature was greater than 38.5 °C. If the antibiotics treatment failed, such as persistent fever without resolution or development of persistent organ failure (POF: organ failure > 48 h defined by the modified Marshall score), a step-up surgical approach consisting of percutaneous catheter drainage, minimal access retroperitoneal necrosectomy, and/or open necrosectomy would be attempted [19, 20].

### Diagnosis of IPN and SPN

A diagnosis of IPN was established when pancreatic or peripancreatic specimens collected via fine-needle aspiration, drainage, or necrosectomy yielded positive microbial cultures [14]. Sterile pancreatic necrosis (SPN) would be defined as negative culture in pancreatic or peripancreatic specimens during the initial surgical intervention, or as the absence of infection signs upon discharge or follow-up without the necessity of any further surgical intervention [4].

### Statistical analysis

Continuous variables with a normal distribution were reported as the mean ± standard deviation, whereas non-normally distributed data were summarized by the median and interquartile range (IQR). Depending on variable characteristics, group comparisons were conducted using the independent-samples t-test, chi-square test, Fisher’s exact test, or Mann–Whitney U test. McNemar’s test was applied to assess differences in sensitivity and specificity among culture, tNGS, and mNGS. Firth’s bias-reduced multivariable logistic regression was used to identify independent factors for positive tNGS. A two-sided *P* < 0.05 was regarded as statistically significant. Given the exploratory nature of this study and the number of hypothesis tests performed, no formal multiplicity correction was applied, and all analyses should be interpreted as exploratory. Statistical analyses were carried out with R software (version 4.3.2; Vienna, Austria).

## Results

### Study cohort

**Fig. 1:**
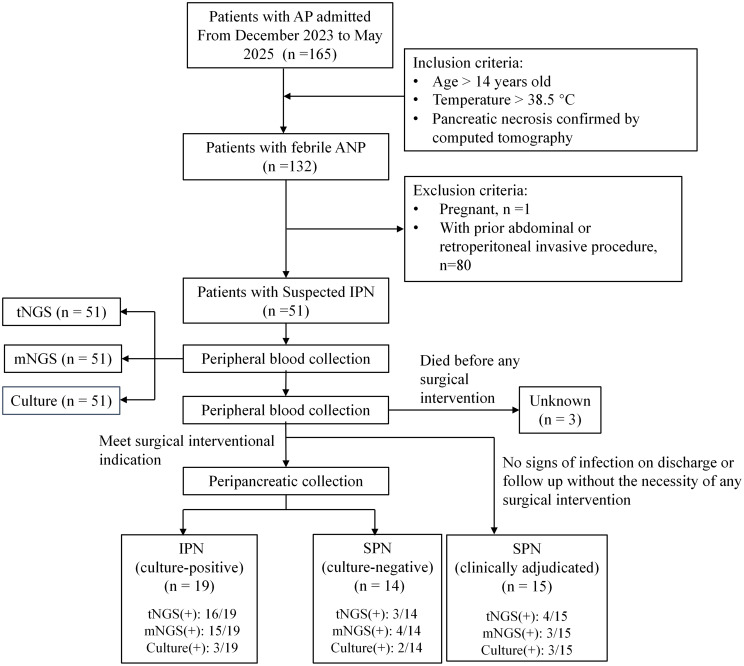
Study flowchart. ANP, acute necrotizing pancreatitis; IPN, infected pancreatic necrosis; SPN, sterile pancreatic necrosis; tNGS, targeted next-generation sequencing; mNGS, metagenomic next-generation sequencing

From December 2023 to May 2025, 51 patients with febrile ANP were consecutively and prospectively enrolled from a pool of 132 ANP patients. The flow chart for patient inclusion was described in Fig. 1. Baseline characteristics, major complications, interventions, and clinical outcomes were summarized in Table 1. Among 51 patients, 41 (80.3%) were male and 10 (19.6%) were female, with a median age of 49 years (38.5–55.0 years). A total of 36 patients were admitted to the ICU with a median (range) length of ICU stay of 6.0 (0.0 to 20.0) days. POF was present in 34.1% of patients. The median time from onset of pancreatitis to blood sampling was 11.0 days (6.5–17.5). IPN was identified in 19 (37.25%), with median time to onset of 15 days (9.3–19.8 days). The types of pancreatic infections included 21.1% of polymicrobial infection, 47.4% of MDRO infection and 21.1% of fungal infection. Three patients (5.9%) who died of irreversible POF or hemorrhage before any surgical intervention were classified as undetermined, as there was no chance of obtaining peripancreatic fluid to confirm the diagnosis of IPN. The remaining 29 patients (56.86%) were classified as SPN, among which 15 patients (51.7%) did not undergo any surgical intervention. The overall in-hospital mortality rate was 23.5% (12/51).

**Fig. 2:**
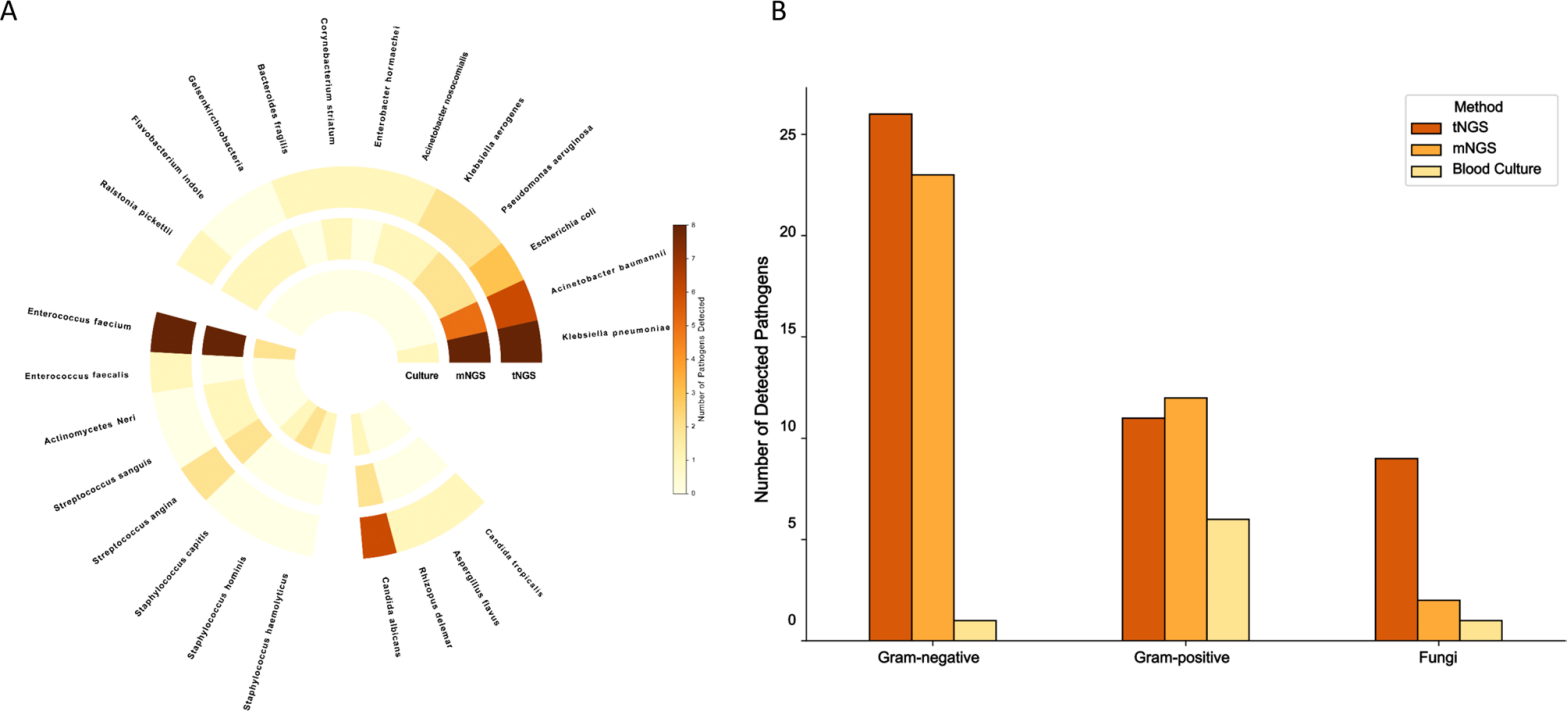
Pathogens detected by different methods. (**A**) Pathogen counts by blood tNGS, mNGS, and culture. (**B**) Distribution of pathogen types across detection methods. tNGS, targeted next-generation sequencing; mNGS, metagenomic next-generation sequencing

**Fig. 3:**
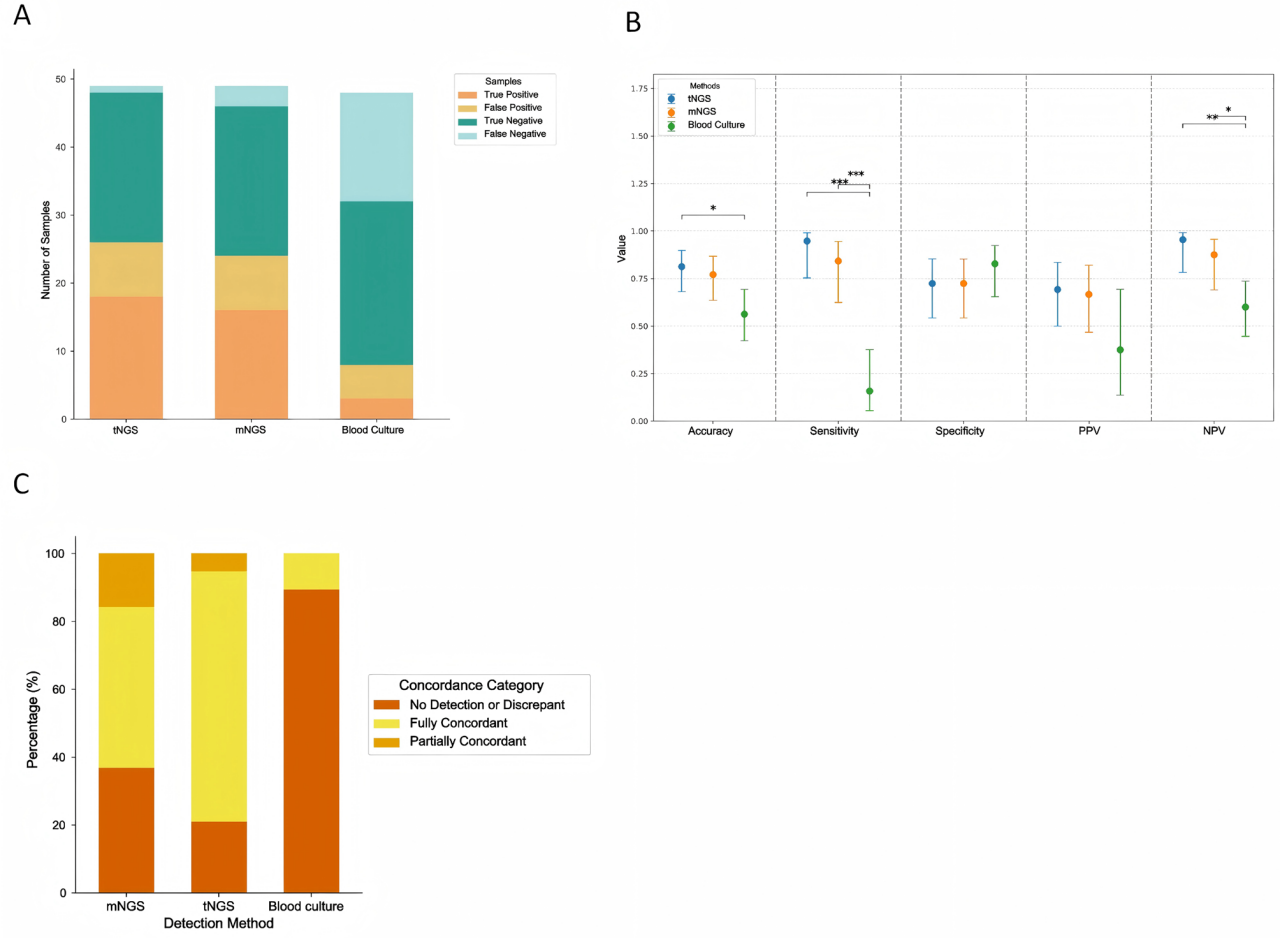
Diagnostic performance of different methods. (**A**) Detection result classification. (**B**) Diagnostic metrics by method. (**C**) Concordance with peripancreatic cultures. tNGS, targeted next-generation sequencing; mNGS, metagenomic next-generation sequencing

**Fig. 4:**
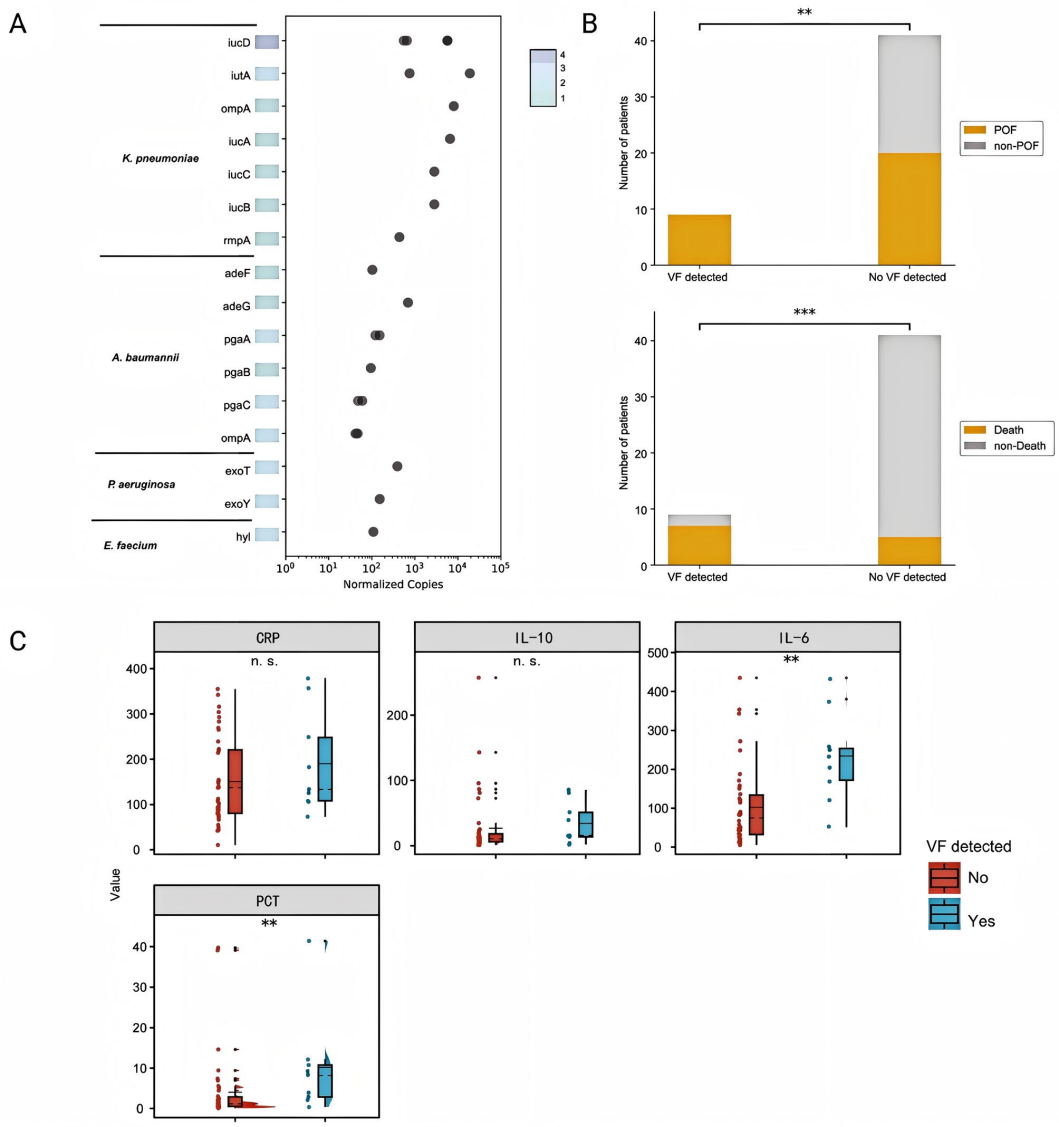
VF detection and clinical correlation. (**A**) VF profiles and normalized signal intensities (per million reads) by pathogen. (**B**) VF detection stratified by severity and mortality. (**C**) Inflammatory markers with or without VF. VF, virulence factor. POF, persistent organ failure

**Fig. 5:**
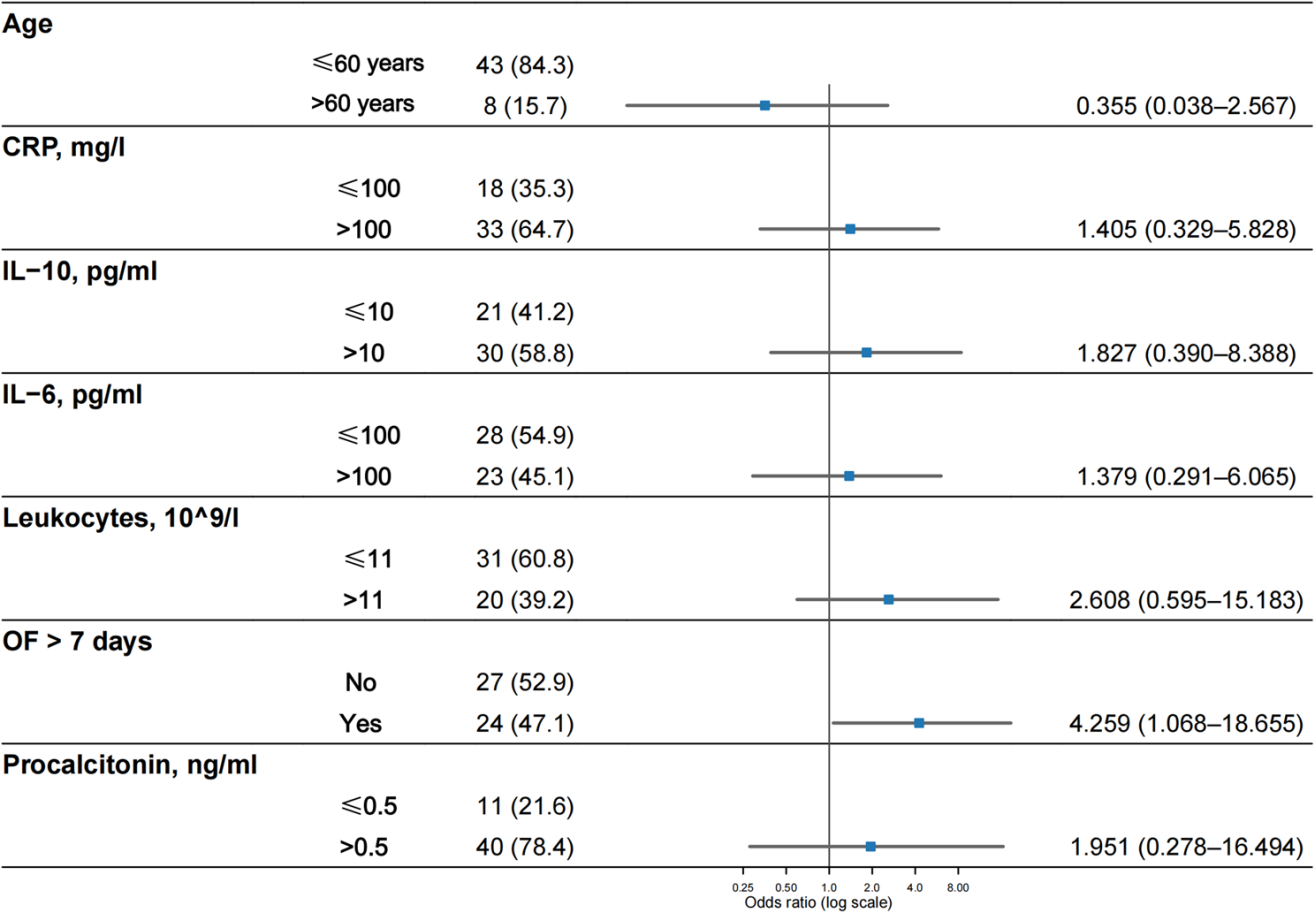
Predictors of pathogen detection by tNGS. IL-6, interleukin-6; IL-10, interleukin-10; CRP, c-reactive protein; OF, organ failure

**Table 1:** Clinical characteristics of the study population

Variable	Level	Overall
Number of patients		51
Age, year		49 (38.5–55)
Gender (%)	Female	10 (19.61)
	Male	41 (80.39)
Etiology (%)	Alcoholic	2 (3.92)
	Biliary	21 (41.18)
	Hypertriglyceridemia	25 (49.02)
	Other	3 (5.88)
Severity (%)	Moderate severe pancreatitis	11 (21.57)
	Severe pancreatitis	40 (78.43)
Leukocytes, 10^9^/l		13.7 (9.75–18.4)
IL-10, pg/ml		12.95 (5.912–23.99)
IL-6, pg/ml		86.18 (45–172)
CRP, mg/l		137 (83.61-222.012)
Procalcitonin, ng/ml		1.274 (0.54–3.405)
Air bubble sign (%)	Negative	47 (92.16)
	Positive	4 (7.84)
Presence of parenchymal necrosis (%)	Parenchymal necrosis	36 (70.6)
	EXPN only	15 (29.4)
Positive blood culture (%)	Negative	42 (82.35)
	Positive	9 (17.65)
Positive blood mNGS (%)	Negative	25 (49.02)
	Positive	26 (50.98)
Positive blood tNGS (%)	Negative	23 (45.10)
	Positive	28 (54.90)
Days from onset of pancreatitis to blood collection, days		6.5 (11-16.5)
Days from fever onset to blood collection, days		3 (1-6.5)
Empirical antibiotics usage (%)	No	6 (11.76)
	Yes	45 (88.24)
	Ceftazidime	7 (13.21)
	Celoperazone/Sulbaclam	12 (22.64)
	Piperacillin/Tazobactam	10 (18.87)
	Meropenem	22 (41.51)
	Etapenem	9 (16.98)
ICU admission (%)	No	15 (29.41)
	Yes	36 (70.59)
Length of ICU stay, days		8 (0–20)
Step up (%)		19 (100.00)
Intestinal fistula (%)	No	47 (92.16)
	Yes	4 (7.84)
Intra-abdominal hemorrhage (%)	No	43 (84.31)
	Yes	8 (15.69)
Pancreatic fistula (%)	No	49 (96.08)
	Yes	2 (3.92)
IPN (%)	IPN	19 (37.25)
	SPN	29 (56.86)
	Unknown	3 (5.88)
Polymicrobial infection (%)	No	15 (78.95)
	Yes	4 (21.05)
MDRO infection (%)	No	10 (52.63)
	Yes	9 (47.37)
Fungal infection (%)	No	15 (78.95)
	Yes	4 (21.05)
Death (%)	No	39 (76.47)
	Yes	12 (23.53)

Data are presented as n (%) or median (IQR)

ANP, acute necrotizing pancreatitis; IL-6, interleukin-6; IL-10, interleukin-10; CRP, c-reactive protein; IPN, infected pancreatic necrosis; EXPN, extrapancreatic necrosis without pancreatic parenchymal necrosis; mNGS, metagenomic next-generation sequencing; tNGS, targeted next-generation sequencing; MDRO, multidrug-resistant organism

### Detection of pathogens and diagnostic efficacy

The overall positive rate of blood tNGS (54.9%) was significantly higher than that of blood culture (17.7%) (*P* < 0.05). The positive rate of tNGS was similar to that of mNGS (51.0%) (*P* = 0.683). More numbers (46 vs. 8 and 46 vs.37) of pathogens were reported by tNGS than blood culture and mNGS. Nine species of fungi were detected by tNGS, which was more than that was achieved with mNGS and culture methods. tNGS also had the tendency to detect more Gram-negative bacteria. (Fig. 2 and Table S3)

Diagnostic performance was evaluated using the reference standard defined in the methodology. Diagnostic performance metrics were compared across all three methods. Sensitivity was highest in blood tNGS at 0.947 (95% CI: 0.754–0.991), followed by blood mNGS at 0.842 (95% CI: 0.624–0.945), while blood culture showed markedly lower sensitivity at 0.158 (95% CI: 0.055–0.376). Conversely, specificity was highest in blood culture at 0.828 (95% CI: 0.655–0.924), whereas both tNGS and mNGS demonstrated a specificity of 0.724 (95% CI: 0.543–0.853). In terms of overall accuracy, blood tNGS (0.813; 95% CI: 0.681–0.898) and mNGS (0.771; 95% CI: 0.635–0.867) outperformed blood culture (0.563; 95% CI: 0.423–0.693). The positive predictive values (PPV) for tNGS, mNGS, and blood culture were 0.692, 0.667, and 0.375, respectively. The negative predictive values (NPV) were 0.955 for tNGS, 0.875 for mNGS, and 0.600 for blood culture. The differences in sensitivity and accuracy between tNGS and blood culture were statistically significant (*P* < 0.001), while comparisons between tNGS and mNGS did not reach statistical significance. (Figure 3A and B and Table S4) A subgroup analysis using peripancreatic culture and a ≤ 72 h sensitivity analysis yielded consistent diagnostic patterns, as presented in Table S5 and S6. In both subgroup analyses, sequencing-based methods continued to outperform blood culture.

### Clinical characteristics of patients classified as “Unknown”

Three patients were categorized as “Unknown” because no peripancreatic sampling was performed and therefore no definitive microbiologic reference was available. Two patients (Cases 2 and 48) had pathogens detected by at least one modality (tNGS, mNGS, or blood culture), accompanied by markedly elevated inflammatory biomarkers and persistent multiple-organ dysfunction, making infection highly suspected. The remaining patient (Case 17) had negative results across all three microbiologic tests, but experienced rapid hemorrhagic and orgaic deterioration, and infection could not be ruled out. (Table S7)

### Pathogen concordance between blood NGS and peri-pancreatic specimen culture

The tNGS achieved a pathogen concordance rate of 78.9% (15/19) with peripancreatic cultures in patients with IPN, significantly higher than that of blood culture [10.5% (2/19), *P* < 0.05]. Among concordant cases (*n* = 15), tNGS completely covered culture results in 14 and showed partial overlap in one. The congruence rate of mNGS results [63.2% (12/19)] was lower than tNGS but not statistically significant (*P* > 0.05). (Fig. 3A)

### Concordance between blood tNGS results and extra-pancreatic infection

Among the 28 patients with positive blood tNGS, 26 patients underwent culture of other specimens including sputum and urine, as shown in Table S8. Among the 15 patients with positive sputum cultures, 13 cases showed that blood tNGS covered the corresponding pathogens identified in the sputum cultures. The G or GM test was positive in 8 cases, and tNGS detected fungi in all cases. After considering symptoms, 7 patients were diagnosed with probable fungi infection. Considering the results of peripancreatic and extra-pancreatic infections, the concordance rate of pathogen detection in 28 cases with positive tNGS results was 89.2% (25/28).

### Turnaround time of blood culture, mNGS, and tNGS

The turnaround times for blood tNGS, blood mNGS, and blood culture were 27.2 (24.0–29.3), 43.5 (38.5–49.2), and 163.8 (159.1–174.4) hours, respectively. Blood tNGS yielded faster results than both blood culture and blood mNGS (*P* < 0.001).

### Antimicrobial-resistance (AMR) gene detection

Antimicrobial-resistance (AMR) determinants were detected in seven patients with available tNGS data. Carbapenemase genes were identified in *A. baumannii* and *K. pneumoniae*, including *KPC-77* (four cases), *KPC-2* (one case), and an *OXA* (one case). Extended-spectrum β-lactamase genes (*CTX-M-65* and *SHV-12*) were detected in one *K. pneumoniae* sample. The *vanA* gene was detected in two patients with *E. faecium*. Among cases with paired peripancreatic cultures, these genotypic findings showed qualitative agreement with the corresponding phenotypic susceptibility profiles. A full list of AMR markers and matched phenotypes is provided in Table 2.

**Table 2:** AMR genes detected by tNGS and matched phenotypic susceptibilities

Case	Species detected by blood tNGS	AMR gene (s) detected	Peripancreatic culture & AST
P4	*A. baumannii*	*KPC-77*	/
P6	*K. pneumoniae; Candida spp.*	*KPC-77*	*K. Pneumoniae* (susceptible only to tigecycline)
P12	*K. pneumoniae*,* P. aeruginosa*,* Enterococcus spp.*,* E. coli*,* A. baumannii*,* Corynebacterium*	*KPC-77*	*P. aeruginosa; K. pneumoniae* (susceptible only to tigecycline); *E. faecium; E. gallinarum; Candida albicans*
P18	*A. baumannii*	*OXA*	*A. Baumannii* (susceptible only to tigecycline)
P31	*K. pneumoniae*	*CTX-M-65*; *KPC-2; SHV-12*	*K. Pneumoniae* (susceptible only to tigecycline)
P41	*E. faecium*	*vanA*	*E. Faecium* (vancomycin-resistant)
P44	*E. faecium; A. baumannii; C. tropicalis *	*vanA*	*E. faecium* (vancomycin-resistant); *A. baumannii; C. tropicalis*

AMR, antimicrobial resistance; AST, antimicrobial susceptibility testing; tNGS, targeted next-generation sequencing; ESBL, extended-spectrum β-lactamase; KPC, Klebsiella pneumoniae carbapenemase; CTX-M, cefotaximase; SHV, sulfhydryl variable; OXA, oxacillinase; vanA, vancomycin resistance gene A

### Virulence factors (VF) and prognosis

In this study, we analyzed the probability of tNGS in virulence identification. After sequencing, 17 VF of 15 kinds were detected for the four pathogens (*K. pneumoniae*,* A. baumannii*,* P. aeruginosa*,* and E. coli*), with a median normalized copy number of 438 per milliliter (IQR 103–2841) (Fig. 4A). In the correlation analysis between VF genes detected and disease severity among patient, patients with VF genes presented a greater proportion of POF (100.0% vs. 57.1%, *P* = 0.015) and mortality (77.7% vs. 11.9%, *P* < 0.001). (Fig. 4B) Meanwhile, procalcitonin and IL-6 levels were also higher in the patients with VF genes (procalcitonin: *P* = 0.008; IL-6: *P* = 0.008). No significant differences were found in the levels of CRP or IL-10 between the two groups (CRP: *P* = 0.294; IL-10: *P* = 0.212). Similar results were found in a subgroup analysis when analyzing patients with positive tNGS. (Table S9)

### Determinants of pathogen detection efficiency by tNGS

To evaluate the factors impacting the detection of causative or potentially causative pathogens by tNGS, we conducted a multivariate logistic regression to calculate odds ratios (ORs), as illustrated in Fig. 5. The results showed that the tNGS positivity rate increased in patients who had OF for more than 7 days at the time of sampling (OR = 4.259; 95% CI: 1.068–18.655; *P* = 0.040), and no differences were found in other subgroups. Although there was a trend suggesting that the subgroup with elevated procalcitonin might have a higher detection potential, there was no significant difference.

## Discussion

tNGS has emerged as an advanced diagnostic technique for detecting infections [21, 22]. While several studies have evaluated the diagnostic performance of tNGS in the context of other infections [16, 23, 24], there is lack of prospective studies that investigate the clinical applicability of this diagnostic method in AP. In this research, we performed a prospective assessment of diagnosing IPN through tNGS. The findings indicated that tNGS showed superior performance to blood cultures and comparable accuracy to mNGS in detecting pathogens and matching peripancreatic cultures. The findings also inform several practice-relevant considerations in IPN.

IPN remains a critical diagnostic challenge, which is associated with high mortality rates of up to 30% [16]. According to IAP/APA guidelines, systemic infection markers and laboratory abnormalities often lack specificity for infected necrosis, while imaging signs, such as gas within peripancreatic enhancement, may be present but remain indeterminate. Fine-needle aspiration is invasive and may yield false-negative cultures Its guidance for therapy has not been conclusively shown to improve outcomes [25]. In contrast, blood‑based mNGS offers a noninvasive modality that could detect circulating pathogens earlier than radiologic changes or invasive sampling, thus narrowing diagnostic uncertainty in patients with systemic infection. However, due to the interference of host DNA and low biomass of circulating pathogens, the applications of mNGS for IPN have faced significant limitations. Furthermore, owing to the complexity of data processing and analysis, high cost, and difficulty to reduce turnaround time, mNGS applications for IPN has encountered limitations. Targeted pathogen enrichment is an emerging approach, offering unprecedented precision and depth in genomic analysis [21].In most clinical scenarios, the spectrum of causative pathogens is relatively predictable. When appropriately designed, tNGS panels can offer comparable diagnostic performance to mNGS while maintaining greater efficiency and clinical applicability [26, 27]. Moreover, one of the key innovations in target enrichment is its ability to multiplex samples prior to enrichment, significantly reducing costs and increasing throughput. Thus, compared to mNGS, tNGS has fewer data burdens and simplified data interpretation, facilitating a lower cost and faster turnaround time. The enhanced analytical sensitivity of tNGS enables the detection of low-burden or transient pathogens that might be overlooked by traditional culture methods or mNGS, especially in patients who have previously undergone antibiotic treatment. However, it is important to note that some background signals may also originate from trace amounts of environmental or commensal DNA.

Our study demonstrated that blood tNGS exhibited comparable accuracy and sensitivity to blood mNGS. Moreover, tNGS demonstrated superior performance compared to mNGS in detecting fungi and Gram-negative bacteria, which are frequently identified in IPN. Some organisms detected exclusively by tNGS but not confirmed by culture or mNGS may reflect its higher sensitivity, particularly for low-abundance or transient bacteremia. However, the possibility of background or false-positive signals cannot be excluded. Chen et al. demonstrated that broad-spectrum tNGS detected the majority of pathogens identified by both mNGS (96.3%, 446/463) and culture (91.2%, 103/113), while also capturing a substantial number of additional organisms [24]. These findings were consistent with our results. By using probe-based capture to enrich pathogen sequences, tNGS likely increases the signal-to-noise ratio, enabling detection of organisms that mNGS may miss. It is widely acknowledged that tNGS significantly improves the identification of fungi, viruses, and bacteria by utilizing probe hybridization enrichment [8]. In our study, tNGS demonstrated superior enrichment for fungal pathogens compared to both mNGS and culture, consistent with findings from recent reports [8, 10]. This disparity highlights the limitations of conventional diagnostic methods in identifying invasive fungal infections, likely due to the fastidious growth requirements of fungi and prior antibiotic exposure that suppresses bacterial proliferation, thereby masking the clinical suspicion of fungal superinfection. To enhance interpretability, discrepant tNGS-only results were evaluated with clinical, imaging, and microbiologic data. Although no strict thresholds were predefined, standard laboratory quality control measures minimized background signals. Additionally, several tNGS-only detections were corroborated by positive G/GM tests or concordant isolates obtained from peripancreatic or extra-pancreatic cultures. Recent evidence indicated that peripancreatic Candida infections did not elevate mortality rates in patients with IPN [28]. In contrast, candidemia was linked to significantly higher mortality and has been identified as an independent predictor of death [29]. These findings suggest that tNGS may support early fungal detection and timely antifungal therapy, although its impact on survival requires further study.

In this study, VF genes detected by tNGS were associated with adverse clinical outcomes in patients with AP. Patients with VF genes exhibited significantly higher rates of mortality and POF. Detection of VF genes was associated with significantly elevated systemic inflammatory biomarkers, including IL-6 and procalcitonin, suggesting a potential mechanistic link between microbial virulence and the host inflammatory response. K. pneumoniae and A. baumannii were the main pathogens detected in this study. While previous studies have established a correlation between these pathogens and poor prognosis in IPN [30], the mechanisms underlying their pathogenicity, as well as the clinical implications of specific subtypes, remain insufficiently characterized. VF genes were co-detected in 8 samples with K. pneumoniae in our study, mainly frequently located on large virulence plasmids, which can be horizontally transferred among K. pneumoniae strains, contributing to the rapid dissemination of hypervirulence (iucA, iucB, icuC, iucD, and iutA) [31]. OmpA, a major outer membrane protein, is highly conserved among Enterobacteriaceae and contributes to the attenuation of the host immune response [32]. These genes are often associated with hypervirulent K. pneumoniae strains, which are increasingly recognized for their ability to cause severe community-acquired infections, such as liver abscesses and pneumonia [31]. Biofilm formation, facilitated by genes (pgaA, pgaB, and pgaC), provides A. baumannii with a protective niche against host immune defenses and antimicrobial agents, significantly enhancing its persistence in clinical settings [33]. Beyond its prognostic associations, VF gene profiling may enhance early risk stratification when integrated with clinical and laboratory parameters. Future research could investigate the application of VF signatures by tNGS in machine learning-based models to facilitate individualized management strategies in IPN. However, due to our comparatively small sample size, this result should be interpreted with caution.

Traditionally, AP mortality has been characterized by two distinct peaks: an early phase driven by systemic inflammatory response syndrome and a late phase associated with IPN [34, 35]. However, infection and organ failure constitute interconnected processes, each amplifying the other and contributing to progressive clinical deterioration [34, 35]. POF may serve as a clinical surrogate for underlying infection in patients with ANP. Our findings support this association by linking POF to the detection of pathogens via tNGS. Clinically, this underscores the importance of maintaining a high index of suspicion for infection in patients whose organ dysfunction persists beyond the first week of illness without signs of resolution. In such scenarios, the application of advanced diagnostic tools such as tNGS may facilitate timely identification of a previously occult infectious etiology. Overall, the observed relationship between sustained organ failure and systemic infection reinforces current guideline recommendations to vigilantly monitor for infection in patients with severe or critical AP, and highlights the potential of NGS to improve early pathogen detection and guide intervention [36].

As an observational study, we were not able to determine whether tNGS-guided identification altered therapeutic decisions or improved clinical outcomes. Current evidence on NGS-guided management in IPN remains limited. A recent retrospective study evaluating mNGS in fine-needle aspirates reported improved microbial yield but no difference in patient survival [37]. This discrepancy may be attributed to several factors, including the timing of mNGS implementation, the severity of underlying conditions, and the presence of comorbidities that complicate treatment efficacy. Compared to mNGS, tNGS offers advantages in turnaround time, cost, and data complexity, which may enhance its compatibility with real-world clinical workflows. Nonetheless, indiscriminate use of NGS has emerged as a concern, particularly in the absence of clearly defined indications and testing windows. Overutilization not only increases healthcare expenditures but may also lead to unnecessary downstream interventions. In our cohort, blood tNGS yielded a higher positivity rate among patients with persistent organ dysfunction and clinical suspicion of infection, suggesting this subgroup may represent a rational target population for future diagnostic use. Prospective studies are warranted to determine whether early, indication-driven use of tNGS can support antimicrobial stewardship and improve clinical outcomes in IPN.

This study has several limitations. First, it was conducted at a single tertiary center with a relatively limited sample size, which may restrict the generalizability of the findings. Second, as an observational cohort, it did not evaluate whether tNGS-guided pathogen detection influenced treatment decisions or improved clinical outcomes such as ICU stay, mortality, or duration of antimicrobial therapy. Third, although tNGS offers potential advantages in turnaround time and cost compared with mNGS, formal cost-effectiveness analyses were not performed, and implementation feasibility may vary across institutions depending on sequencing infrastructure, personnel, and clinical demand. Fourth, the interpretation of tNGS-only results remains challenging in the absence of culture or mNGS confirmation, highlighting the need for integrated interpretation frameworks that combine sequencing results with clinical, radiologic, and biomarker data. Finally, several diagnoses of SPN were based on clinical adjudication rather than microbiological confirmation, which introduces the potential for misclassification bias. Nevertheless, the overall stability of results in both the culture-verified subgroup and the timing-restricted sensitivity analysis reinforces the robustness of our conclusions. Future research should aim to validate the diagnostic performance and clinical utility of blood tNGS in multicenter settings with larger, prospectively enrolled cohorts. Interventional studies incorporating predefined treatment protocols and outcome endpoints are warranted to determine whether early tNGS-guided antimicrobial therapy improves clinical outcomes and supports antimicrobial stewardship in patients with suspected IPN.

## Conclusion

In summary, tNGS provides diagnostic accuracy comparable to mNGS with faster turnaround, and may offer prognostic insights through virulence factor detection.

## Supplementary Information

Below is the link to the electronic supplementary material.

Supplementary Material 1

Supplementary Material 2

## Data Availability

The data are available at (https://ngdc.cncb.ac.cn/ Reference number: PRJCA044938) The data supporting the findings of this study are available upon reasonable request from the corresponding author.

## Abbreviations

AP: Acute pancreatitis
ANP: Acute necrotizing pancreatitis
CRP: C-reactive protein
CT: Computed tomography
GM: Galactomannan
G test: β-D-glucan test
ICU: Intensive care unit
IL-6: Interleukin-6
IL-10: Interleukin-10
IPN: Infected pancreatic necrosis
IQR: Interquartile range
MDRO: Multidrug-resistant organism
mNGS: Metagenomic next-generation sequencing
NGS: Next-generation sequencing
NPV: Negative predictive value
OR: Odds ratio
POF: Persistent organ failure
PPV: Positive predictive value
SOFA: Sequential Organ Failure Assessment
SPN: Sterile pancreatic necrosis
STROCSS: Strengthening the Reporting of Cohort Studies in Surgery
tNGS: Targeted next-generation sequencing
VF: Virulence factor
