# Supplementary material for: Clinical evaluation of probe capture-based targeted next-generation sequencing in suspected infected pancreatic necrosis: a prospective pilot diagnostic study
Source: BMC Infect Dis. 2025 Dec 29;26:215. doi: 10.1186/s12879-025-12441-w (PMC12859840; doi:10.1186/s12879-025-12441-w)
Supplement: Supplementary file 1 — Supplementary Material 1 [file 12879_2025_12441_MOESM1_ESM.docx]

**Table S1.** Diagnostic workflows and a relative cost framework for blood culture, tNGS, and mNGS

|  | **Workflow steps** | **Direct Relative Cost**  **(Mainly reagents & consumables)** | **Indirect Relative Cost**  **(Mainly labor hours & time-related resource usage)** | **Comprehensive Relative Cost** | **Notes** |
| --- | --- | --- | --- | --- | --- |
| Blood Culture | sample reception → nucleic-acid extraction → library preparation → targeted enrichment → sequencing → bioinformatic analysis → report generation | 1 | 1 | 1 | Long instrument occupancy cycle |
| tNGS | sample reception → nucleic-acid extraction → library preparation → whole-genome sequencing → bioinformatic analysis → report generation | 4.0-5.0 | 0.4-0.6 | 2.9-3.7 | Lower amortized sequencing cost; no long-term instrument occupancy cost |
| mNGS | sample reception → isothermal incubation → positive-signal alert → subculture and organism identification → report release, acknowledging inherent batching and long instrument-occupancy periods | 7.0-8.0 | 0.8-1.0 | 5.1-5.9 | Highest cost for library preparation and sequencing chips |

a.All indicators take the cost of blood culture as the benchmark value (1.0).

b.The comprehensive relative cost is calculated using a weighted average (direct cost weight: 70%, indirect cost weight: 30%).

**Table S2.** Virulence-factor (VF) panel included in the tNGS workflow

| **Species** | **Virulence factors** |
| --- | --- |
| Escherichia coli (extraintestinal pathogenic) | pic, hlyC, hlyA, hlyB, hlyD, iucC, iucB, iucA, iroB, ompA |
| Acinetobacter baumannii | pgaA, pgaB, pgaC, pgaD, ompA, plc1, adeG, adeH |
| Anaplasma phagocytophilum | ompA |
| Bordetella pertussis | ptxA, ptxB, ptxD, ptxE, ptxC |
| Corynebacterium diphtheriae | tox |
| Francisella tularensis | ompA |
| Klebsiella pneumoniae | rmpA2, iroB, iucA, iucB, iucC, iucD, iutA, ompA, rmph, magA |
| Pseudomonas aeruginosa | exoS, exoT, exoY, exoU, algD, lasA, lasB, plcH |
| Staphylococcus aureus | eta, etb, lukF-PV, lukS-PV, tsst-1, seb, hlb, hly/hla, spa, sea, seh, sec, lukE, lukD, see |
| Streptococcus pyogenes | emm, speA |
| Listeria monocytogenes | hly |
| Mycoplasma pneumoniae | MPN_RS02090 |
| Streptococcus pneumoniae | cbpA/pspC, lytA, psaA, pspA, ply, nanA |
| Campylobacter jejuni | cdtA, cdtB, cdtC |
| Clostridioides difficile | cdtA, cdtB |
| Escherichia coli (diarrheagenic) | aggR, eae, bfpA, cdtC, stx1A, stx1B, stx2A, stx2B, hlyA, hlyB, hlyC, hlyD, pic, pet, east1, estⅠa, eltA, eltB, toxB, east1, agg3A, agg3B, agg3C, agg3D |
| Shigella spp. | pic, icsA/virG, ipgB1, icsB, mxiG, virA, virB, icsP/sopA, ospG, ipaH7.8, ipaH1.4, ipaH2.5, ipaH0722, ipaH |

Table S3. Distribution of pathogens identified by blood tNGS, mNGS and Culture.

| **Pathogen** | **Method** | | |
| --- | --- | --- | --- |
|  | **tNGS** | **mNGS** | **Blood Culture** |
| *Gram-Negative Bacteria* |  |  |  |
| Klebsiella pneumoniae | 8 | 8 | 1 |
| Acinetobacter baumannii | 6 | 5 | 0 |
| Escherichia coli | 3 | 2 | 0 |
| Pseudomonas aeruginosa | 2 | 2 | 0 |
| Klebsiella aerogenes | 2 | 1 | 0 |
| Acinetobacter nosocomialis | 1 | 1 | 0 |
| Enterobacter hormaechei | 1 | 0 | 0 |
| Corynebacterium striatum | 1 | 1 | 0 |
| Bacteroides fragilis | 1 | 0 | 0 |
| Gelsenkirchnobacteria | 0 | 1 | 0 |
| Flavobacterium indole | 0 | 1 | 0 |
| Ralstonia pickettii | 1 | 1 | 0 |
| *Gram-Positive Bacteria* |  |  |  |
| Enterococcus faecium | 8 | 8 | 2 |
| Enterococcus faecalis | 1 | 0 | 0 |
| Actinomycetes Neri | 0 | 1 | 0 |
| Streptococcus sanguis | 0 | 1 | 0 |
| Streptococcus angina | 2 | 2 | 0 |
| Staphylococcus capitis | 0 | 0 | 1 |
| Staphylococcus hominis | 0 | 0 | 2 |
| Staphylococcus haemolyticus | 0 | 0 | 1 |
| *Fungi* |  |  |  |
| Candida albicans | 6 | 2 | 1 |
| Rhizopus delemar | 1 | 0 | 0 |
| Aspergillus flavus | 1 | 0 | 0 |
| Candida tropicalis | 1 | 0 | 0 |

Data are presented as n.

tNGS, targeted next-generation sequencing; mNGS, metagenomic next-generation sequencing;

Table S4. Diagnostic performance between blood tNGS, mNGS, and culture.

| Method | Metric | Mean | CI Lower | CI Upper |
| --- | --- | --- | --- | --- |
| tNGS | Accuracy | 0.813 | 0.681 | 0.898 |
| tNGS | Sensitivity | 0.947 | 0.754 | 0.991 |
| tNGS | Specificity | 0.724 | 0.543 | 0.853 |
| tNGS | PPV | 0.692 | 0.500 | 0.835 |
| tNGS | NPV | 0.955 | 0.782 | 0.992 |
| mNGS | Accuracy | 0.771 | 0.635 | 0.867 |
| mNGS | Sensitivity | 0.842 | 0.624 | 0.945 |
| mNGS | Specificity | 0.724 | 0.543 | 0.853 |
| mNGS | PPV | 0.667 | 0.467 | 0.820 |
| mNGS | NPV | 0.875 | 0.690 | 0.957 |
| Blood Culture | Accuracy | 0.563 | 0.423 | 0.693 |
| Blood Culture | Sensitivity | 0.158 | 0.055 | 0.376 |
| Blood Culture | Specificity | 0.828 | 0.655 | 0.924 |
| Blood Culture | PPV | 0.375 | 0.137 | 0.694 |
| Blood Culture | NPV | 0.600 | 0.446 | 0.737 |

tNGS, targeted next-generation sequencing; mNGS, metagenomic next-generation sequencing; PPV, positive predictive value; NPV, negative predictive value;

Table S5. Diagnostic performance between blood tNGS, mNGS, and culture in the complete-verification subgroup (n = 33).

| **Method** | **Metric** | **Mean** | **CI Lower** | **CI Upper** |
| --- | --- | --- | --- | --- |
| tNGS | Accuracy | 0.818 | 0.656 | 0.914 |
| tNGS | Sensitivity | 0.842 | 0.624 | 0.945 |
| tNGS | Specificity | 0.786 | 0.524 | 0.924 |
| tNGS | PPV | 0.842 | 0.624 | 0.945 |
| tNGS | NPV | 0.786 | 0.524 | 0.924 |
| mNGS | Accuracy | 0.758 | 0.59 | 0.872 |
| mNGS | Sensitivity | 0.789 | 0.567 | 0.915 |
| mNGS | Specificity | 0.714 | 0.454 | 0.883 |
| mNGS | PPV | 0.789 | 0.567 | 0.915 |
| mNGS | NPV | 0.714 | 0.454 | 0.883 |
| Blood Culture | Accuracy | 0.455 | 0.298 | 0.62 |
| Blood Culture | Sensitivity | 0.158 | 0.055 | 0.376 |
| Blood Culture | Specificity | 0.857 | 0.601 | 0.96 |
| Blood Culture | PPV | 0.6 | 0.231 | 0.882 |
| Blood Culture | NPV | 0.429 | 0.265 | 0.609 |

tNGS, targeted next-generation sequencing; mNGS, metagenomic next-generation sequencing; PPV, positive predictive value; NPV, negative predictive value;

**Table S6.** Diagnostic performance of tNGS, mNGS, and blood culture in patients sampled within ≤72 hours (n = 19)

| **Method** | **Accuracy** | **Sensitivity** | **Specificity** | **PPV** | **NPV** |
| --- | --- | --- | --- | --- | --- |
| tNGS | 0.895 | 0.8 | 1 | 1 | 0.818 |
| mNGS | 0.842 | 0.8 | 0.889 | 0.889 | 0.8 |
| Blood culture | 0.421 | 0.1 | 0.778 | 0.333 | 0.438 |

Table S7. Clinical characteristics of the three patients with “unknown”

| **Case** | **Age**  **(years)** | **Key inflammatory markers** | **tNGS** | **mNGS** | **Blood culture** | **Cause of death** |
| --- | --- | --- | --- | --- | --- | --- |
| P2 | 51 | PCT 10.1 ng/mL; CRP 243 mg/L; IL-6 200 pg/mL; IL-10 13.8 pg/mL; WBC 20×10⁹/L | K. pneumoniae | K. pneumoniae | Negative | MODS |
| P17 | 44 | PCT 6.28 ng/mL; CRP 239 mg/L; IL-6 248 pg/mL; WBC 25×10⁹/L | Negative | Negative | Negative | Hemorrhagic shock + MODS |
| P48 | 18 | PCT 93.9 ng/mL; CRP 150 mg/L; IL-6 20 pg/mL; IL-10 12.9 pg/mL; WBC 18×10⁹/L | Candida; K. pneumoniae | Negative | Candida | MODS |

Table S8. Consistency between blood tNGS results and microbes detected by culture in other specimens or G/GM test

| Patient | **Blood tNGS** | **Peri-pancreatic culture** | **Extra-pancreatic spcimen culture** | **G/GM test** |
| --- | --- | --- | --- | --- |
| 1 | *Klebsiella pneumoniae;  Acinetobacter baumannii* | *-* | Sputum：*Klebsiella pneumoniae* | Neg |
| 2 | *Klebsiella pneumoniae* | *-* | Sputum：*Klebsiella pneumoniae* | - |
| 3 | *Streptococcus anginosus; Acinetobacter baumannii* | *-* | Sputum：Neg | - |
| 4 | *Acinetobacter baumannii* | *-* | Sputum：*Acinetobacter baumannii* | - |
| 6 | *Klebsiella pneumoniae;  Candida albicans* | *Klebsiella pneumoniae* | Urine： *Candida albicans* | GM Pos |
| 7 | *Escherichia coli* | *Escherichia coli* | Sputum：*Klebsiella pneumoniae* | - |
| 8 | *​Enterococcus faecium* | *​Enterococcus faecium* | Sputum：*Acinetobacter baumannii* | - |
| 9 | *Rhizopus delemar​* | Neg | Sputum：*Acinetobacter baumannii* | GM Pos |
| 10 | *Acinetobacter baumannii; Enterococcus faecalis; Enterococcus faecium; ​Aspergillus flavus​* | Neg | - | G & GM Pos |
| 11 | *Candida albicans* | Neg | Sputum：Neg | G Pos |
| 12 | *Klebsiella pneumoniae; Enterococcus faecalis; Pseudomonas aeruginosa; Escherichia coli; Acinetobacter baumannii; Corynebacterium striatum* | *Pseudomonas aeruginosa;  Klebsiella pneumoniae;  Enterococcus faecalis;  Candida albicans* | Sputum：Acinetobacter baumannii;  Pseudomonas aeruginosa | Neg |
| 18 | *Acinetobacter baumannii* | *Acinetobacter baumannii* | Sputum：Neg | Neg |
| 21 | *Escherichia coli;  Bacteroides fragilis; ​ Enterococcus faecium* | *Enterococcus faecium* | Sputum：Neg | - |
| 26 | *Klebsiella aerogenes* | *Klebsiella aerogenes* | Sputum：Klebsiella aerogenes | - |
| 27 | *Pseudomonas aeruginosa* | Neg | Sputum：*Acinetobacter baumannii* | - |
| 29 | *Candida albicans* | *-* | Sputum：Neg | G Pos |
| 31 | *Klebsiella pneumoniae* | *Klebsiella pneumoniae* | Sputum：*Klebsiella pneumoniae* | Neg |
| 32 | *Klebsiella aerogenes* | Neg | Sputum：Klebsiella aerogenes | - |
| 34 | *Klebsiella pneumoniae* | *Klebsiella pneumoniae* | Sputum：*Klebsiella pneumoniae;  Pseudomonas aeruginosa* | - |
| 35 | *Enterococcus faecium​* | *Candida glabrata* | Sputum：Neg | - |
| 36 | *Hospital Acinetobacter* | *Streptococcus mitis​* | Sputum：Neg | Neg |
| 41 | *Enterococcus faecium* | *Enterococcus faecium* | Sputum：Neg | Neg |
| 42 | *Candida albicans* | *Candida albicans* | Sputum：Neg | G Pos |
| 44 | *Enterococcus faecium;  Acinetobacter baumannii; ​ Candida tropicalis​* | *Enterococcus faecium;  Acinetobacter baumannii; ​ Candida tropicalis​* | Sputum：*Acinetobacter baumannii* | G Pos |
| 45 | *Enterococcus faecium* | Neg | Sputum：*Burkholderia cepacia*;  Candida albicans* | Neg |
| 47 | *Ralstonia pickettii;  Candida albicans* | *-* | - | G Pos |
| 48 | *Klebsiella pneumoniae;  Enterobacter hormaechei;  Candida glabrata​* | *-* | Sputum：*Klebsiella pneumoniae* | GM Pos |
| 51 | *Streptococcus anginosus* | *Acinetobacter baumannii;  Enterococcus faecium* | Sputum：*Streptococcus anginosus* | GM Pos |

tNGS, targeted next-generation sequencing; mNGS, metagenomic next-generation sequencing;

*colonization

Table S9. Subgroup analysis of patients with positive tNGS Based on virulence factor detection status

| Variables | Level | Overall | No VF detected | VF detected | *P*-value |
| --- | --- | --- | --- | --- | --- |
| n |  | 28 | 19 | 9 |  |
| IL-6 |  | 131 (76.163-190.795) | 90.5 (52.695-153.745) | 234.7 (172-253.7) | **0.003** |
| IL-10 |  | 15.34 (10.123-56.508) | 14.8 (9.665-53.665) | 15.68 (13.81-51.1) | 0.646 |
| procalcitonin |  | 2.713 (0.648-7.05) | 1.533 (0.581-5.205) | 8.16 (2.867-10.772) | **0.032** |
| CRP |  | 136.52 (85.848-219.838) | 140.04 (74.89-216.5) | 133 (108-248) | 0.242 |
| POF (%) | No | 6 (21.43) | 6 (31.58) | 0 (0.00) | 0.159 |
| POF (%) | Yes | 22 (78.57) | 13 (68.42) | 9 (100.00) |  |
| Death (%) | No | 17 (60.71) | 15 (78.95) | 2 (22.22) | **0.014** |
| Death (%) | Yes | 11 (39.29) | 4 (21.05) | 7 (77.78) |  |

Data are presented as n (%) or median (IQR).

VF, virulence factor; IL-6, interleukin-6; IL-10, interleukin-10; CRP, c-reactive protein; POF, persistent organ failure.
