# Supplementary material for: Clinical evaluation of probe capture-based targeted next-generation sequencing in suspected infected pancreatic necrosis: a prospective pilot diagnostic study
Source: BMC Infect Dis. 2025 Dec 29;26:215. doi: 10.1186/s12879-025-12441-w (PMC12859840; doi:10.1186/s12879-025-12441-w)
Supplement: Supplementary file 2 — Supplementary Material 2 [file 12879_2025_12441_MOESM2_ESM.pdf]

STARD Checklist for Reporting Diagnostic Accuracy Studies

Item	STARD requirement	Where reported in manuscript
1	Title identifies as diagnostic accuracy study	Title; Abstract
2	Structured abstract including index test, reference standard, accuracy	Abstract
3	Scientific background, intended use, role of test	Introduction
4	Study objectives	Introduction, last paragraph
5	Study design (prospective/retrospective)	Methods – Study design
6	Eligibility criteria	Methods – Study design
7	Setting, locations, dates	Methods – Study design
8	Participant recruitment method	Methods – Sample collection
9	Sampling method (consecutive?)	Methods – Study design
10	Index test details	Methods – mNGS process; tNGS process
11	Reference standard details	Methods – Sample collection + peripancreatic culture
12	Rationale for reference standard	Discussion – limitations partial verification bias）
13	Definition of positivity (thresholds)	Methods – VF threshold; index test positivity
14	Blinding of test interpreters	Methods
15	Availability of clinical data to assessors	Methods – Laboratory process （implicitly blinded）
16	Handling of indeterminate results	Flowchart figure; Methods – exclusions
17	Handling of missing data	Methods – Statistical analysis
18	Participant flow diagram	Figure 1（STARD-flow revised）
19	Baseline characteristics	Results – Table 1
20	Severity/clinical spectrum	Table 1; Results（clinical characteristics）
21	Index test results	Results – Figures 2, 3
22	Cross-tabulation of test vs reference	Results – Diagnostic metrics; confusion matrices
23	Accuracy estimates + CIs	Results – Figure 3; Supplementary Table
24	Adverse events from testing	N/A (blood-based tests, stated unobtrusive)
25	Study limitations (including verification/selection bias)	Discussion – limitations
26	Clinical implications, generalisability	Discussion
27	Registration	Methods
28	Full protocol availability	Ethics paragraph
29	Funding	Section added per reviewer request
30	Role of funder	Funding section

STROBE Checklist for Cohort Studies

Item	Requirement	Where reported
1	Title/abstract indicate cohort study	Title; Abstract
2	Background/rationale	Introduction
3	Objectives	Introduction
4	Study design	Methods – first paragraph
5	Setting	Methods – Study design
6	Participants (criteria)	Methods – Study design
7	Variables definition	Methods – sample collection; biomarkers; index test; VF
8	Data sources/measurement	Methods – mNGS; tNGS; culture
9	Bias handling	Discussion – limitations
10	Study size	Participant flow (n=…)
11	Handling quantitative variables	Methods – cutoffs defined（VF ≥60% coverage; biomarkers dichotomized）
12	Statistical methods	Methods – Statistical analysis
13	Participant flow	Figure 1
14	Descriptive data	Table 1
15	Outcome data	Results – IPN/SPN breakdown
16	Main results	Results – Figures 2–4
17	Other analyses	Subgroup analyses (culture-verified, ≤72h sensitivity analysis)
18	Key results summary	Discussion
19	Limitations	Discussion
20	Interpretation	Discussion
21	Generalisability	Discussion
22	Funding	Added per reviewer request
